# Angioimmunoblastic T-Cell Lymphoma after Treatment of Classic Hodgkin Lymphoma: A Case Report

**DOI:** 10.3390/hematolrep15040067

**Published:** 2023-11-25

**Authors:** Ken Tanaka, Hiroaki Miyoshi, Yusuke Yamashita, Ryuta Iwamoto, Yuma Yokoya, Yuichi Tochino, Fumiko Arakawa, Shinobu Tamura, Shin-Ichi Murata, Takashi Sonoki, Koichi Ohshima

**Affiliations:** 1 Department of Pathology, Kurume University School of Medicine, Kurume 8300011, Japan; ken-t@wakayama-med.ac.jp (K.T.); arakawa_fumiko@med.kurume-u.ac.jp (F.A.); ohshima_kouichi@med.kurume-u.ac.jp (K.O.); 2 Department of Hematology/Oncology, Wakayama Medical University, Wakayama 6418509, Japan; yyyamash@wakayama-med.ac.jp (Y.Y.); y-yokoya@wakayama-med.ac.jp (Y.Y.); ytochino@wakayama-med.ac.jp (Y.T.); stamura@wakayama-med.ac.jp (S.T.); sonoki@wakayama-med.ac.jp (T.S.); 3 Department of Diagnostic Pathology, Wakayama Medical University, Wakayama 6418509, Japan; riwamoto@wakayama-med.ac.jp (R.I.); smurata@wakayama-med.ac.jp (S.-I.M.)

**Keywords:** classic hodgkin lymphoma, lymphocyte rich classic hodgkin lymphoma, angioimmunoblastic T-cell lymphoma, secondary non-hodgkin lymphoma

## Abstract

We report a case of a 24-year-old man who developed angioimmunoblastic T-cell lymphoma (AITL) after treatment for refractory lymphocyte-rich classic Hodgkin lymphoma (LR-CHL). This patient was treated with the BV+AVD (brentuximab vedotin, doxorubicin, vinblastine, and dacarbazine) protocol for LR-CHL but progressed before completing chemotherapy. The pathological imaging showed the typical findings of LR-CHL at the first onset and first progression. Rescue chemotherapy and high-dose chemotherapy combined with autologous hematopoietic stem cell transplantation (AHSCT) were performed for refractory LR-CHL, and complete remission was achieved. However, the recurrence was suspected 6 months after AHSCT. The pathological findings of the lymph node biopsy at this time were different from those of the previous two lymph node biopsies, demonstrating findings of AITL. The finding of the immunohistochemical staining and polymerase chain reaction results supported the diagnosis. Although it has been reported that the risk for the development of non-Hodgkin lymphoma after treatment for Hodgkin lymphoma is increased, most are B-cell lymphomas, and few cases of AITL have been reported. AITL is a type of peripheral T-cell lymphoma that generally occurs in middle-aged and elderly people and that rarely occurs in young people. Here, we were able to make an accurate diagnosis by performing re-examination even when recurrence of LR-CHL was suspected. As there are no detailed case reports of AITL developing into secondary non-Hodgkin lymphoma, here we report on an identified case.

## 1. Introduction

Classic Hodgkin lymphoma (CHL) is a malignant lymphoid tumor that originates from B cells and often presents in young people [1,2]. CHL is considered to have a relatively good prognosis among lymphomas. With proper treatment, CHL is expected to have a long-term response of 80% or greater [2,3]. Lymphocyte-rich classic Hodgkin lymphoma (LR-CHL) is a subtype of CHL associated with favorable prognosis [4]. However, the prognosis of relapsed and refractory CHL remains worse than that of pretreatment CHL [5].

Although rare, it has been reported that secondary non-Hodgkin lymphoma (sNHL) may develop after treatment for CHL [6,7,8]. Eichenauer et al. reported that high-grade B-cell lymphoma is the most common type of sNHL and that the proportion of T-cell lymphomas is lower than that of B-cell lymphomas [6]. It has been reported that the characteristics of patients who are prone to develop sNHL include being older, being male, and having an advanced stage of disease at the time of diagnosis of CHL [8].

AITL is a subtype of peripheral T-cell lymphoma (PTCL) with a T follicular helper (TFH) cell phenotype [9]. Pathological findings of AITL are characterized by the presence of clear cells, high endothelial venules (HEVs) proliferation, and follicular dendritic cell (FDC) hyperplasia. However, in some AITL cases, large lymphocytes resembling Hodgkin cells may appear, making it difficult to differentiate it from CHL [9]. 

As there are no detailed case reports of AITL developing into sNHL, here we report on an identified case.

## 2. Case Presentation

A 24-year-old man presented with bilateral lymphadenopathies. During two months of follow-up, the patient’s lymph nodes continued to grow. Hence, a right cervical lymph node biopsy (biopsy 1) was performed. Based on the pathological diagnosis, the patient was diagnosed with lymphocyte-rich classic Hodgkin lymphoma (LR-CHL). The patient was diagnosed with stage IIIA disease by positron emission tomography/computed tomography (PET/CT) and treated with BV+AVD (brentuximab vedotin, doxorubicin, vinblastine, and dacarbazine) protocol. The patient’s lymph nodes temporarily decreased in size but then grew again during the third course of chemotherapy. Progression of LR-CHL was suspected, and a left cervical lymph node biopsy (biopsy 2) was performed. According to the biopsy, the progression of LR-CHL was confirmed. As this case of LR-CHL was considered a relapse/refractory case, the treatment was changed to ICE (ifosfamide, carboplatin, etoposide). After four courses of ICE, complete remission (CR) was achieved, and intensive chemotherapy with autologous hematopoietic stem cell transplantation (AHSCT) was performed. After AHSCT, BV monotherapy was continued. However, multiple systemic lymph node swelling reappeared 6 months after AHSCT. A second relapse of LR-CHL was considered, and a right supraclavicular lymph node biopsy (biopsy 3) was performed. Based on the pathological diagnosis, AITL was diagnosed. We considered starting salvage chemotherapy; however, this patient requested an outpatient follow-up. Hence, the patient is being followed up carefully with outpatient visits every 6 weeks. Every 6 months, it is confirmed by PET/CT or contrast-enhanced CT whether the lymph node lesions have increased or whether new lesions have appeared. Even after 18 months of follow-up, although the multiple systemic lymphadenopathy remains, an enlargement of lymph nodes and symptoms due to malignant lymphoma have not been observed. When AITL progression is suspected, we plan to perform a lymph node biopsy and consider appropriate salvage chemotherapy.

## 3. Materials and Methods

### 3.1. Immunohistochemical Stains

Immunohistochemical (IHC) staining was performed on formalin-fixed paraffin-embedded (FFPE) tissue sections using antibodies against CD3 (Clone F7.2.38; Dako), CD4 (Clone SP35; Ventana), CD30 (Clone Ber-H2; Dako), PAX5 (Clone 1EW; Leica), ICOS (Clone D1K2T; Cell Signaling), PD-1 (Clone NAT105; Abcam), and FDC (Clone CAN 4.2; Dako).

### 3.2. In Situ Hybridization for Epstein–Barr Virus-Encoded RNA (EBER)

Epstein–Barr virus (EBV) was detected using in situ hybridization with a fluorescein-conjugated EBV peptide nucleic acid probe kit (DakoCytomatin, Glostrup, Denmark) by following the manufacturer’s instructions.

### 3.3. Polymerase Chain Reaction (PCR) for the Detection of Immunoglobulin Heavy Chain (IgVH) Rearrangements and T-Cell Receptor Gamma Chain (TCRɤ) Rearrangements

DNA was extracted from FFPE tissues using a commercial kit (KAPA Express Extract Kit, KAPA BIOSYSTEMS, Wilmington, MA, USA). Semi-nested PCR was performed as previously described [10] to analyze clonal IgVH and TCRɤ rearrangements in the sample. Amplified PCR products were electrophoresed on 3% agarose gels, stained with ethidium bromide, and visualized under ultraviolet light. β-actin was used as an internal control. The IgVH and TCRɤ PCR products were estimated to be approximately 250 and 230 bp, respectively.

## 4. Pathological Presentation

### 4.1. Biopsy 1 (Biopsy Specimen of a Cervical Lymph Node at the First Onset)

**Figure 1:**
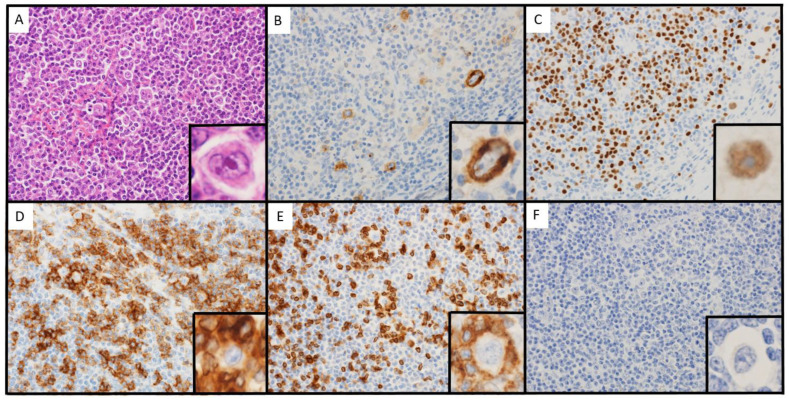
Microscopic findings of biopsy 1. (**A**): hematoxylin-eosin (×400). (**B**): Immunohistochemical (IHC) staining of CD30 (×400). (**C**): IHC staining of PAX5 (×400). (**D**): IHC staining of CD3 (×400). (**E**): IHC staining of PD-1 (×400). (**F**): Epstein–Barr virus-encoded RNA in situ hybridization (×400).

**Figure 2:**
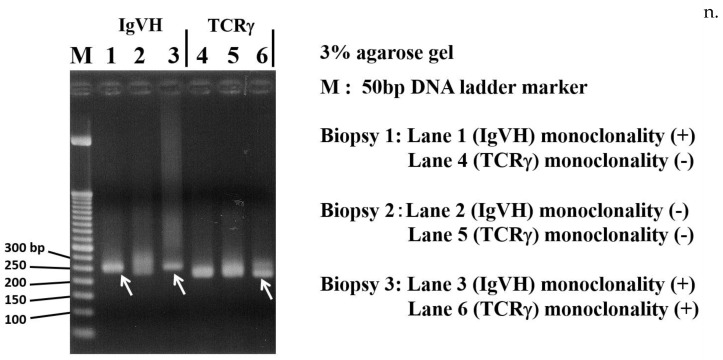
Immunoglobulin heavy chain (IgVH) gene rearrangement and T−cell receptor gamma chain (TCRɤ) gene rearrangement evaluated by polymerase chain reaction. bp: base pair. IgVH: immunoglobulin heavy chain. TCRɤ: T-cell receptor gamma chain.

Histologically, the patient’s cervical lymph node exhibited a nodular growth pattern, and the normal lymph node structure was obscured. Hodgkin/Reed-Sternberg (HRS) cells proliferate sporadically, and lymphocytes with no atypia were observed in the background (Figure 1A). IHC staining showed that the HRS cells expressed CD30 and PAX5 (Figure 1B,C). In addition, CD3-positive and PD-1-positive T-cells formed a rosette around the HRS cells (Figure 1D,E). EBER was negative in the HRS cells (Figure 1F). A band showing a monoclonal IgVH rearrangement was observed by PCR (Figure 2). Based on these findings, the patient was diagnosed with LR-CHL. 

Lanes 1 and 4 are specimens from biopsy 1. A band showing the monoclonality of the IgH gene rearrangement is observed (arrow). Lanes 2 and 5 are specimens from biopsy 2. Monoclonality is not evident for the IgVH and TCRɤ gene rearrangement. Lanes 3 and 6 are specimens from biopsy 3. A band representing monoclonality is evident for both the IgVH gene rearrangement and TCRɤ gene rearrangement (arrow).

### 4.2. Biopsy 2 (Biopsy Specimen of a Cervical Lymph Node at the First Recurrence)

**Figure 3:**
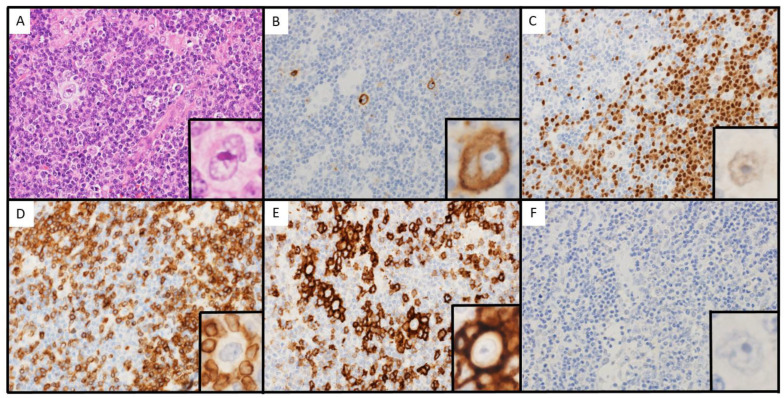
Microscopic findings of biopsy 2. (**A**): hematoxylin-eosin (×400). (**B**): Immunohistochemical (IHC) staining of CD30 (×400). (**C**): IHC staining of PAX5 (×400). (**D**): IHC staining of CD3 (×400). (**E**): IHC staining of PD-1 (×400). (**F**): Epstein–Barr virus-encoded RNA in situ hybridization (×400).

The histological image was similar to that of biopsy 1. HRS cells proliferated, and lymphocytes with no atypia were observed in the background (Figure 3A). IHC staining also showed findings similar to those of biopsy 1 (Figure 3B–F). In addition, monoclonality was not observed for IgVH and TCRɤ rearrangements (Figure 2). Thus, the patient was diagnosed with progression of LR-CHL. 

### 4.3. Biopsy 3 (Biopsy Specimen of a Supraclavicular Lymph Node When the Recurrence of LR-CHL Was Suspected)

**Figure 4:**
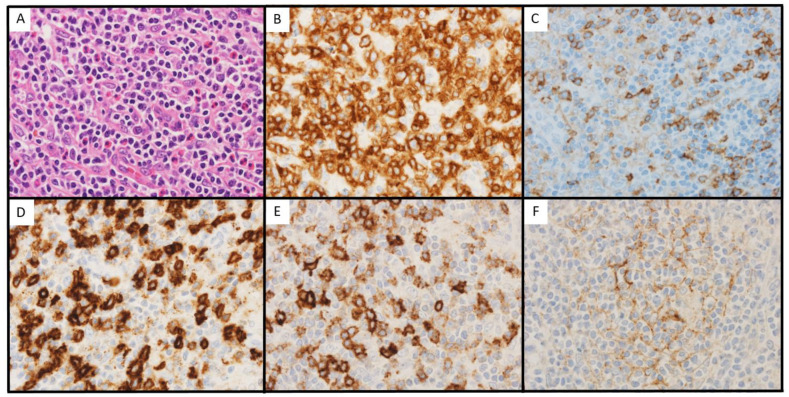
Microscopic findings of biopsy 3. (**A**): hematoxylin-eosin (×600). (**B**): Immunohistochemical (IHC) staining of CD3 (×600). (**C**): IHC staining of CD4 (×600). (**D**): IHC staining of ICOS (×600). (**E**): IHC staining of PD-1 (×600). (**F**): IHC staining of FDC (×600).

The normal structure of the lymph nodes was completely effaced. The proliferation of small-to-medium-sized lymphocytes with clear cytoplasm was observed, and the number of eosinophils and HEVs increased (Figure 4A). Large, atypical cells were also found, whereas typical HRS cells were not. Following IHC stainings, the small-to-medium-sized atypical lymphocytes were positive for CD3 and CD4 (Figure 4B,C). These cells expressed ICOS and PD-1 (Figure 4D,E), and irregular hyperplasia of the FDC meshwork was also observed (Figure 4F). EBER was negative in all cells. A band showing monoclonality was observed for both IgVH and TCRɤ rearrangements (Figure 2). Based on these findings, AITL was diagnosed at this time.

## 5. Discussion

We report the case of a young man who developed AITL soon after treatment for relapsed/refractory LR-CHL. The clinical and pathological findings of the three biopsies are summarized in Table 1.

One point to be discussed is that the patient may have exhibited PTCL from the time of first onset or first progression. Several cases have been reported in which PTCL of TFH cell origin, such as AITL and follicular T-cell lymphoma (FTCL), mimic LR-CHL [11,12,13,14]. These reports suggest that it is important to distinguish between PTCL and LR-CHL based on morphological and IHC findings in addition to molecular cytological findings such as PCR. In biopsies 1 and 2, eosinophil infiltration and HEVs were not evident, and typical HRS cells were observed. In contrast, opposite findings were observed in biopsy 3, and irregular hyperplasia of the FDC was detected. In previous reports, it seems difficult to distinguish between AITL and LR-CHL based on pathological findings alone [11,12,13,14]. However, in this case, biopsy 1 and 2 showed clearly different histological findings from biopsy 3. In addition to these histological findings, a TCRɤ rearrangement was not observed in biopsies 1 and 2. However, in biopsy 3, monoclonality was observed not only in the TCRɤ but also in the IgVH rearrangement, and it has been reported that this feature is found in 25–30% of AITL cases [15,16]. Considering these findings comprehensively, it was considered that AITL was not observed in biopsies 1 and 2 and developed only in biopsy 3.

There are other important points to consider in this case. One notable point is that AITL developed in a young adult even though AITL is generally considered a PTCL that frequently occurs in middle-aged and elderly people [17,18,19]. In previous reports, the median onset of AITL was 64.5–68 years, and 60–75% of AITL patients were over 60 years of age [18,19]. Although previous studies including cases of young people have been reported [16,18], detailed case reports that included histological images, medical treatment, and outcomes after treatment could not be identified in our search. Thus, it is difficult to predict the future clinical course of this patient. Although there are few reports on the causes and risks of AITL in adolescents and young adults, chemotherapy for CHL may have been significantly associated with the onset of AITL in this case.

The AITL onset after CHL treatment is also rare and important to consider. It has been reported that sNHL occurs at a rate of approximately 0.9–1.5% after treatment for HL, and its prognosis is poor compared to that of HL [6,7,8]. T-cell lymphoma is relatively rare, and AITL is reported to occur in 10.5% of T-cell lymphoma and 1.5% of all sNHL cases [8]. As the number of cases of PTCL as sNHLs is small and there are no reports that have been examined in detail, a future accumulation of cases and their analyses are awaited.

The exact cause of this rare clinical course is unknown, but it may be related to refractory CHL. In this case, chemotherapies, such as BV+AVD, ICE and AHSCT, were repeated, and it was considered that these chemotherapies may have caused the deterioration of the patient’s immune function. Combined with this background, AITL may have developed as early as about half a year after treatment for CHL.

Lymph node biopsy was performed each time recurrence was suspected, which led to the elucidation of the pathophysiology of this case. If there is a history of CHL and lymphadenopathy, it is common to consider CHL recurrence. In actual clinical practice, the course of recurrent lymphoma is consistent, and if biopsy is difficult due to the patient’s general condition or tumor site, patients with a recurrence of lymphoma may be treated based on the clinical course and laboratory findings. It is necessary to consider the possibility of sNHL, especially in patients with a history of multiple lines of chemotherapy. Treatment may be significantly different between CHL and sNHL, and we believe that re-examination should be actively performed even when CHL recurrence is suspected.

## 6. Conclusions

Here, we report the case of a young man who developed AITL after treatment for LR-CHL. There is a rare possibility of other lymphomas developing after treatment with CHL, as in this case. The choice of treatment depends greatly on the diagnosis. Even if recurrence is suspected, re-biopsy should be performed as frequently as possible. In addition, this case underlines that the evaluation of IgVH gene rearrangement and TCRɤ gene rearrangement is helpful for diagnosis.

## Figures and Tables

**Table 1:** The clinical and pathological findings of the three biopsies.

		Biopsy 1	Biopsy 2	Biopsy 3
Clinical finding	Symptom	Lymphadenopathy	Lymphadenopathy	Lymphadenopathy
	Localization of lesion	Cervix, supraclavicular fossa, mediastinum, inguen	Cervix	Cervix, supraclavicular fossa, mediastinum, axilla, inguen, para-aorta, liver hilum
	Stage (Ann Arbor)	IIIA	IA	IIIA
Pathological finding	Pathological finding (HE)	Typical HRS cell (+), HEVs (−) eosinophil infiltration (−)	Typical HRS cell (+), HEVs (−) eosinophil infiltration (−)	Typical HRS cell (−), HEVs (+) eosinophil infiltration (+)
	Pathological finding of HRS cells	CD3 (−), CD20 (−), CD30 (+) PAX5 (+), EBER (−)	CD3 (−), CD20 (−), CD30 (+), PAX5 (+), EBER (−)	No typical HRS cell
	Pathological finding of T cells	Atypia (−). Rosette formation on HRS cells (+). IHC: CD3 (+), CD4>CD8, PD-1 (partial +), ICOS (partial +)	Atypia (−). Rosette formation on HRS cells (+). IHC: CD3 (+), CD4>CD8, PD-1 (partial +), ICOS (partial +)	Atypia (+). IHC: CD3 (+), CD4 (+), CD8 (−), PD-1 (+), ICOS (+), CD10 (−), BCL6 (−). Irregular FDC (+)
	Gene rearrangement (PCR)	IgVH (+), TCRɤ (−)	IgVH (−), TCRɤ (−)	IgVH (+), TCRɤ (+)
	Diagnosis	Lymphocyte-rich classic Hodgkin lymphoma	Lymphocyte-rich classic Hodgkin lymphoma	Angioimmunoblastic T-cell lymphoma
Clinical course	Treatment	BV + AVD	ICE + AHSCT → BV monotherapy	Watchful waiting
	Therapeutic effect	PR → PD	CR	SD
	Time from first onset to recurrence (day)	First onset	108 days	482 days

HE—hematoxylin and eosin stain; HRS—Hodgkin/Reed–Sternberg; HEVs—high endothelial venules; EBER—Epstein–Barr virus-encoded RNA in situ hybridization; IHC—immunohistochemistry; PD-1—programmed death receptor-1; ICOS—inducible T-cell co-stimulator; FDC—follicular dendritic cell; PCR—polymerase chain reaction; IgVH—immunoglobulin heavy chain; TCRɤ—T-cell receptor gamma chain; BV—brentuximab vedotin; AVD—doxorubicin, vinblastine, dacarbazine; ICE—ifosfamide, carboplatin, etoposide; AHSCT—autologous hematopoietic stem cell transplantation; PR—partial response; PD—progressive disease; CR—complete remission; SD—stable disease.

## Data Availability

The data used and analyzed during the current study are available from the first author or the corresponding author on reasonable request.
